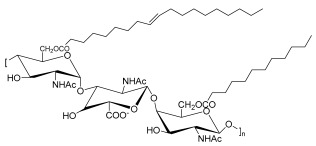# Exploring Biosurfactants as Antimicrobial Approaches

**DOI:** 10.3390/ph17091239

**Published:** 2024-09-19

**Authors:** Madalena Lourenço, Noélia Duarte, Isabel A. C. Ribeiro

**Affiliations:** Research Institute for Medicines (iMed.ULisboa), Faculty of Pharmacy, Universidade de Lisboa, Avenida Prof. Gama Pinto, 1649-003 Lisboa, Portugal; madalenalourenco1@edu.ulisboa.pt

**Keywords:** biosurfactants, antimicrobial, contact killing, antimicrobial release, antifouling, structure–activity relationship

## Abstract

Antibacterial resistance is one of the most important global threats to human health. Several studies have been performed to overcome this problem and infection-preventive approaches appear as promising solutions. Novel antimicrobial preventive molecules are needed and microbial biosurfactants have been explored in that scope. Considering their structure, these biomolecules can be divided into different classes, glycolipids and lipopeptides being the most studied. Besides their antimicrobial activity, biosurfactants have the advantage of being biocompatible, biodegradable, and non-toxic, which favor their application in several areas, including the health sector. Often, the most difficult infections to fight are associated with biofilm formation, particularly in medical devices. Strategies to overcome micro-organism attachment are thus emergent, and it is possible to take advantage of the antimicrobial/antibiofilm properties of biosurfactants to produce surfaces that are more resistant to the deposition/attachment of bacteria. Approaches such as the covalent bond of biosurfactants to the medical device surface leading to repulsive physical–chemical interactions or contact killing can be selected. Simpler strategies such as the absorption of biosurfactants on surfaces are also possible, eliminating micro-organisms in the vicinity. This review will focus on the physical and chemical characteristics of biosurfactants, their antimicrobial activity, antimicrobial/antibiofilm approaches, and finally on their structure–activity relationship.

## 1. Introduction

The widespread use of antibiotics, whether in a clinical environment, animal feeding, or even agriculture, has triggered bacterial resistance, posing challenges in preventing and treating bacterial infections [1,2]. According to the World Health Organization (WHO), over 50% of bacterial infections are becoming resistant to treatment, increasing morbidity and mortality rates [3]. Comparing the data from 2017 and 2020 in 87 countries, the level of resistance of bacterial infections such as bloodstream infections due to *Escherichia coli* and *Salmonella* spp. increased by at least 15%. Moreover, some urinary tract infections caused by *E. coli* are resistant to first-line (e.g., ampicillin) and second-line (e.g., fluoroquinolones) drugs [4]. Additionally, the Global Antimicrobial Resistance and Antimicrobial Use Surveillance System (GLASS) pointed out that high levels of resistance in bacteria are a recurrent cause of sepsis in hospital settings [4].

**Figure 1:**
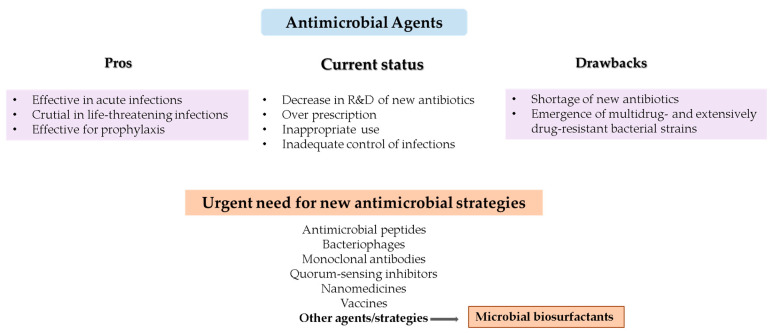
Major advantages and drawbacks of current antimicrobial therapy and alternative strategies to overcome the shortage of new antibiotics and antimicrobial resistance.

The vast majority of antibiotics were discovered between 1929 and 1962 [5]. Since the 2000s, only oxazolidinones, such as cycloserine and linezolid, and the cyclic lipopeptide daptomycin entered the market [5]. Over the last few decades, most of the pharmaceutical industries have decreased their interest in the research and development of antimicrobial drugs because they do not assure market expansion and revenues [6]. Several strategies have been proposed to address the shortage of new antibiotics and, more importantly, to overcome bacterial resistance (Figure 1). These alternative approaches include the development of antimicrobial peptides, bacteriophages and their encoded endolysins, monoclonal antibodies, quorum-sensing inhibitors, nanomedicines, vaccines, and antibiofilm agents [7,8,9].

The formation of biofilms on medical devices is a major challenge in bacterial infection control. Biofilms are networks of microbial populations attached to a surface and covered by an extracellular matrix of proteins, lipids, and polysaccharides, creating a barrier that protects bacteria from penetration by antimicrobial agents [8,10]. A bacterium in a biofilm can be up to a thousand times more tolerant to these external agents than in the planktonic form [7,11]. To survive in hostile environments, biofilms not only act as a physical barrier against exogenous stress, but also reduce the metabolic rates of the cells. As a result, biofilm-associated infections are difficult to eradicate and pose a risk to the prevalence of persistent chronic diseases [12]. Therefore, targeting the initial attachment of bacteria may be a promising strategy to prevent biofilm formation and further infection. Several passive and active methods have been developed to impart antimicrobial properties to medical devices by preventing the attachment of micro-organisms. Passive approaches include the modification of the physical or chemical properties of surfaces aimed at repelling bacteria (e.g., changing the surface’s hydrophilic nature). On the other hand, active methods involve coating surfaces with antimicrobial agents capable of interfering with microbial cells [13]. Different coatings have been proposed, including those which release antibacterial agents (e.g., antibiotics, antiseptics, nitric oxide, and silver), or act by contact (e.g., quaternary ammonium compounds, chitosan, antimicrobial peptides, and enzymes) [13,14]. However, research should not only focus on these compounds but should continue to search for new solutions. A possible solution may involve the use of biosurfactants, which are molecules of biological origin that have been gaining attention as an alternative to synthetic surfactants due to their particular properties, which include lower toxicity and higher biodegradability and biocompatibility, among others.

This review presents and discusses the physical and chemical characteristics of biosurfactants, their antimicrobial activity, antimicrobial/antibiofilm approaches, and finally their structure–activity relationship.

The literature search was carried out using PubMed, Web of Science, and Science Direct, covering the last two decades of research. An appropriate set of keywords was employed (e.g., biosurfactants, antimicrobial activity, antibacterial activity, antifouling, contact killing, etc.). Only peer-reviewed research or review publications in English were considered. The authors exhaustively selected and screened the literature based on quality, accuracy, and relevance to the aim of the review. The software Mendeley Reference Manager (version 1.19.8, 2020) was employed to manage the references and eliminate duplicates.

## 2. Biosurfactants: Classes and Physicochemical Properties

Surfactants are amphiphilic molecules that tend to disperse at the interface between liquid phases of different polarities, lowering surface and interfacial tensions, and promoting detergency, emulsification, lubrication, solubilization, and phase dispersion. They are highly demanded compounds used in almost every industrial area, including food, pharmaceuticals, cosmetics, agriculture, textile and fibers, petroleum and oil, plastics, resins, and detergents [15,16,17,18,19].

Currently, the majority of commercially available surfactants are synthetic and derived from petroleum-based sources, having a negative environmental impact due to their toxicity and poor biocompatibility and biodegradability [15,20,21]. In this regard, it is very important to find alternatives that are both environmentally friendly and compliant with the United Nations Sustainable Development Goals [22]. Various naturally occurring substances with surfactant properties have been isolated and identified from several sources, including plants, bacteria, yeast, or filamentous fungi [15,16,17]. Natural surfactants can be gathered in two major groups based on their origin. First-generation biosurfactants are compounds isolated or chemically produced from plant- or animal-based sources, such as saponins, sugar esters, alkyl polyglucosides, and alkanolamines. Second-generation biosurfactants, also called microbial biosurfactants (mBSs) or green surfactants are molecules entirely produced from renewable microbial resources or by a biological process (biocatalysis or fermentation). Among second generation biosurfactants are microbial glycolipids such as sophorolipids and rhamnolipids and lipotpetides such as surfactin that will be, among others, further discussed [19,23].

Compared to their synthetic counterparts, mBSs present several advantages due to their biodegradable nature and renewable production methods [24]. Their production yield depends on several factors, such as the sources and ratio of carbon and nitrogen, the presence of minerals, temperature and pH, stirring speed, and incubation time. Furthermore, industrial waste can be used to produce biosurfactants, highlighting the sustainability of this approach [25,26,27].

**Figure 2:**
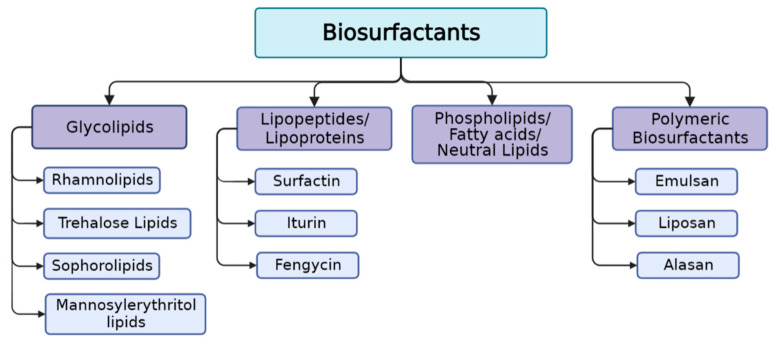
Classification of microbial biosurfactants according to their chemical structure.

Microbial biosurfactants can be classified according to their molecular weight and chemical structure (Figure 2 and Table 1). Low-molecular-weight mBSs include glycolipids, lipopeptides, and phospholipids, and are more effective in decreasing interfacial tension and surface tension at the oil–water and air–water interfaces. High-molecular-weight mBSs include polymeric compounds such as polysaccharides, proteins, or combined forms of lipoproteins and lipopolysaccharides, often named bioemulsifiers because of their ability to stabilize oil-in-water emulsions [28,29].

Glycolipids are the most studied class of low molecular weight mBSs, and include rhamnolipids, trehalolipids, sophorolipids, and mannosylerythritol lipids. Several applications of glycolipids in the pharmaceutical area have been reported [30]. This class of molecules has a hydrophilic moiety (polar head group), usually glucose, galactose, xylose, or rhamnose, covalently linked (via an ether or ester bond) to a lipidic hydrophobic chain [31,32]. The hydrophobic moiety can be a long chain of saturated or unsaturated aliphatic acids or aliphatic hydroxyl acids. Glycolipids preserve the hydrophilic–hydrophobic balance, managing to reduce the interfacial tension between the cell and an external environment, controlling the growth, reproduction, and colonization of microbial communities (i.e., biofilms) [16,26,31].

Rhamnolipids (RLs), one of the most studied classes, consist of (*L*)-rhamnose units with a glycosidic bond to one or more saturated/unsaturated β-hydroxy fatty acid chains ranging from 8 to 24 carbon atoms [16,31,33]. These compounds are mainly produced by *Pseudomonas aeruginosa* and exist mostly in two forms: mono-rhamnolipids, with one rhamnose unit, and di-rhamnolipids, with two sugar units linked together through a ∝-1,2-glycosidic bond [34,35]. Microbial fermentation results in a variety of rhamnolipid congeners and related compounds, which differ in chain length, degree of unsaturation of the fatty acid chains, and in the number of rhamnose molecules. The specific types and quantities of these congeners depend on the strains of micro-organisms involved in the production process [36] as well as the carbon source used and culture conditions [20].

Trehalolipids are constituted by a non-reducing disaccharide trehalose linked through an ester bond to an α-branched-β-hydroxy acid, among which mycolic acid is predominant [37]. These metabolites are mainly produced by *Rhodococcus erythropolis*, *Nocardia*, *Mycobacteria*, and *Corynebacteria* species. Trehalose biosurfactants exhibit antimicrobial activity against Gram-positive bacteria and some pathogenic fungi [38,39].

Sophorolipids (SLs) have a sophorose sugar moiety covalently linked to a long-chain hydroxyl fatty acid containing 16 or 18 carbon atoms and can occur either in the lactonic or acidic form. Several yeast species can produce this type of glycolipid, *Candida bombicola* being the most reported. Regarding their antimicrobial activity, lactonic forms present higher antibacterial activity while the acidic ones present higher antiviral activity [16,31,40,41].

Finally, mannosylerythritol lipids (MELs) are a type of glycolipid biosurfactant containing 4-O-β-*D*-mannopyranosyl-erythritol or 1-O-β-*D*-mannopyranosyl-erythritol (hydrophilic moiety) bonded to a fatty acid. Among other species, they can be produced by *Pseudozyma antartica* or *Pseudozyma aphidis* and can exist in different forms (MEL-A, MEL-B, MEL-C, and MEL-D) depending on the substituent groups (Table 1) [35,42,43,44].

Lipopeptides are the second most important class of mBS and have also been reported to present antifungal, antibacterial, antiviral, and antitumor activities [45,46,47]. They are cyclic peptides that are linked to an acylated fatty acid molecule. Among lipopeptides are surfactins, iturins, and fengycin, which can be produced extracellularly by numerous micro-organisms, particularly *Bacillus subtilus* [16,48]. Surfactins are the most well-studied lipopeptides, consisting of seven long-chain hydrophobic amino acids linked to a fatty acid chain by a lactone bond. Considered one of the most effective biosurfactants available, it has been reported to have antibacterial, antifungal, antimycoplasmal, antiviral, and antitumoral activities [49].

Phospholipids are the primary components of microbial cell membranes. Due to their tiny head group, phospholipids reduce interfacial tension by forming microemulsions, which are vital for medicinal applications. *Acinetobacter radioresistens* is the primary source of phosphatidylethanolamine, which represents the most significant phospholipid among BSs [16,50,51,52].

Polymeric biosurfactants are surface active molecules with a high molecular weight which can be produced by different microbial genera, including *Pseudomonas*, *Arthrobacter*, *Bacillus*, *Acinetobacter*, *Halomonas*, and *Candida* [53]. The best-known compounds in this group are emulsan, alasan, and liposan. Typically, high-molecular-weight BSs are better emulsion stabilizers when compared to those of low molecular weight. Emulsan can work as an emulsifier for hydrocarbons in water at very low concentrations [26,27,50]. Liposan provides very stable emulsions (oil in water) because it coats the oil droplets, and thus is useful in the cosmetic and food industries [45].

Compared to synthetic surfactants, mBSs offer several advantages, including lower or no toxicity, higher biodegradability, specificity, stability and production yield under adverse conditions of temperature, pH, and ionic strength [15,54,55]. For example, when comparing biosurfactants to synthetic surfactants, the former can withstand concentrations of NaCl as high as 10%, but 2% is sufficient to inactivate the latter [15,50,54]. In addition, mBSs can be produced from renewable substrates and resources (e.g., food, agriculture, or agro-industrial waste residues), contributing to economic and environmental sustainability [15,27,53,56].

One of the most important properties of a surfactant is the ability to reduce surface and interfacial tension. Surface tension can be defined as the work required to increase the size of the surface of a phase. Since it measures the work per unit of area or the force per wetted length, surface tension is expressed in mN/m [57,58]. The intermolecular forces between two liquids that do not mix, such as water and oil, produce interfacial tension. For instance, when interfacial tension in a mixture of water and hexadecane is reduced from 40 to 1 mN/m by adding a surfactant, it is possible to classify that surfactant as good [16,54]. A surfactant is considered effective when it reduces the surface tension of water from 72 to 35 mN/m [16,54,59]. Another important parameter is the critical micelle concentration (CMC) which is the concentration of a surfactant in a bulk phase, above which aggregates of surfactant molecules, i.e., micelles, begin to form. When this concentration value is reached, the adsorption of the molecules through the interface is completed. For values above the CMC, there are no relevant changes in surface activity, as it does not affect the number of surfactant monomers in the solution, only the structure of micelles [60]. Therefore, the effectiveness and efficiency of a surfactant is evaluated by its ability to decrease surface and interfacial tensions and its CMC value. In general, BSs have higher efficiency and effectiveness than synthetic surfactants, since lower surface tension can be achieved with lower quantities [18,26,27].

Considering all of the benefits of mBSs as surface-active molecules, several studies have focused on accessing their efficacy and efficiency depending on the producing organism and the growth medium selected (Table 2).

**Table 2:** Effectiveness and efficiency of some biosurfactants considering the producing micro-organism and the growth medium.

Micro-Organism	Growth Medium	mBS	Properties		Ref.
			CMC (mg/L)	γCMC (mNm^−1^) ^a^	
*P. aeruginosa*		Rhamnolipids			[61,62]
	Soybean oil waste	R2C10C10 (pure) ^b^	110.0	28.8	
	Soybean oil waste	M6 (mixture) ^c^	230.0	27.3	
	Soybean oil waste	M7 (mixture) ^d^	150.0	26.8	
	Frying oil waste	RL47T2 (mixture) ^e^	108.0	32.8	
	Sugarcane molasses + corn steep liquor	Mono-rhamnolipids	50.0	25.9	[63]
		Di-rhamnolipids	15.0	33.5	
	Sugarcane molasses + corn steep liquor + NaCl (875 mM)	Mono-rhamnolipids	25.0	25.9	[64]
		Di-rhamnolipids	15.0	31.7	
*S. bombicola*	GPY seed medium supplemented oleic acid or borage oil	Sophorolipids			[65]
		L-C18:0 diacetylated	29.2	35.7	
		L-C18:1 diacetylated	31.2	36.3	
		L-C18:2 diacetylated	35.0	38.5	
		L-C18:3 diacetylated	39.1	38.8	
*B. subtilis*	Sucrose, peptone, yeast extract, MgSO_4_·7H_2_O, Na_2_HPO_4_·12H_2_O, KH_2_PO_4_	Surfactins	250.0	27.9	[48,66]
	Mineral Salt Solution with:				[67]
	Glucose	Surfactins	325.1	29.2	
	Glycerol	Surfactins	154.1	29.7	
	Lactose	Surfactins	65.3	30.7	

^a^ Surface tension at CMC; ^b^ L-rhamnosyl-rhamnosyl-3-hydroxydecanoyl-3-hydroxydecanoate; ^c^ (R_1_C_10_C_10_ + R_2_C_10_C_12_ + R_1_C_10_C_12_ + R_1_C_12:1_C_10_ + R_1_C_12:2_ + R_1_C_8:2_); ^d^ (R_2_C_10_C_10_ + R_1_C_10_C_10_ + R_2_C_10_C_12_ + R_1_C_10_C_12_ + R_1_C_12:1_C_10_ + R_1_C_12:2_ + R_1_C_8:2_, R_2_ is L-rhamnosyl; R_1_ is H or 3-hydroxydecanoate; ^e^ Rha-Rha-C_8_-C_10_+Rha-C_10_-C_8_ + Rha-Rha-C_8_-C_12:1_ + Rha-Rha-C_10_-C_10_ + Rha-Rha-C_10_-C_12:1_ + Rha-C_10_-C_10_ + Rha-Rha-C_10_-C_12_ + Rha-C_10_-C_12:1_ + Rha-Rha-C_12:1_-C_12_ + Rha-Rha-C_10_-C_14:1_ + Rha-C_10_-C_12_.

In addition to the ability to reduce surface tension, biosurfactants also have several biological activities, including antimicrobial properties [68,69], which has triggered the interest in their use as antimicrobial alternatives aimed at preventing infections.

## 3. Biosurfactants’ Antimicrobial Activity

**Figure 3:**
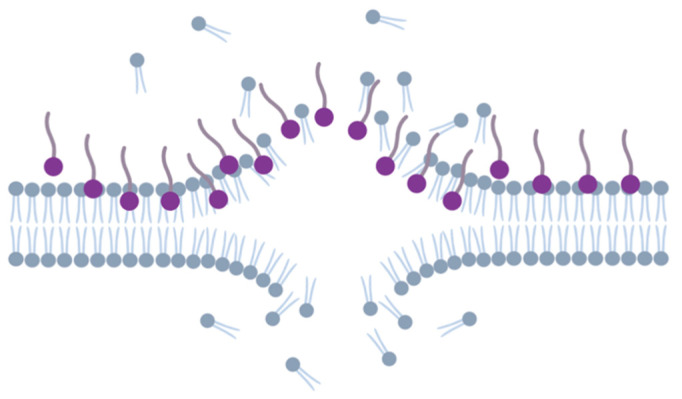
Mechanism of action of antimicrobial biosurfactants towards bacteria. Microbial biosurfactants (in purple) binding to the membrane surface (in grey) will lead to structural alterations with consequent rupture and cell lyses.

The antimicrobial activity of many biosurfactants arises from their ability to disrupt the lipid bilayer of membranes, ultimately resulting in cell death [32,36]. This process of membrane rupture starts with the mBS binding to the membrane surface, inducing structural alterations. Subsequently, reorganization takes place within the membrane, culminating in its rupture and cell lysis (Figure 3) [7,70,71,72].

Negatively charged bacterial membranes (due to peptidoglycan and lipopolysaccharides) will attract positively charged mBSs and an enhancement of their antimicrobial effect is expected. In contrast, neutral biosurfactants demonstrate less affinity for membrane binding. Nevertheless, they still have the ability to eradicate bacteria and hinder biofilm formation [73]. For rapidly acting on the membrane rather than targeting specific sites/targets, mBSs may act as an alternative to fight emerging antibiotic resistance [6]. Generally, mBSs exhibit greater efficacy against Gram-positive bacteria compared to Gram-negative bacteria because they possess an outer membrane comprised of endotoxin (lipopolysaccharide), which operates as a protective barrier [58].

The concern regarding infections extends not only to bacterial infections but also to the proliferation of fungal infections. Biosurfactants may also play a role in fungi-related infection prevention in animals and plants, namely as biocides [59]. Under European legislation, a biocidal product is defined as a substance intended to eradicate, repel, or neutralize harmful organisms [60]. Considering their role in eliminating living organisms, these biocides may inherently carry risks to human and environmental health. An alternative to these products involves selecting biocides derived from natural sources, such as biosurfactants [61].

Antimicrobial activity has been mostly described for glycolipid and lipopeptide mBSs. Various types of glycolipids have been identified for their antimicrobial properties, including rhamnolipids produced by *Pseudomonas aeruginosa*, sophorolipids produced by *Starmerella bombicola* [74,75], and mannosylerythritol lipids (MEL-A and MEL-B) produced by *Candida antarctica* [62,76]. Lipopeptides, such as surfactins, fengycin, iturin, and polymyxin B produced by the *Bacillus* genus, also exhibit antimicrobial activity. Additionally, there are lesser-known antimicrobial lipopeptides such as lichenysin and pumilacidin, produced by *Bacillus licheniformis* and *Bacillus pumilus*, respectively [62,63]. The antimicrobial potential of these mBSs as well as the conditions selected for testing their antimicrobial activity are summarized in Table 3.

**Table 3:** Antimicrobial activity of biosurfactants (BSs) produced by different micro-organisms and testing methods used.

BS	Micro-Organism and Method	Results	Ref.
RLs	*P. aeruginosa* AT10 MIC: dilution method	Antibacterial activity against Gram-negative bacteria: *A. faecalis* (32 µg/mL), *B. bronchiseptica* (128 µg/mL), *E. coli* (32 µg/mL), *S. thyphinurium* (128 µg/mL), *S. marcescens* (16 µg/mL). Antibacterial activity against Gram-positive bacteria: *A. oxidans* (16 µg/mL), *B. subtilis* (64 µg/mL), *M. luteus* (32 µg/mL), *M. phlei* (16 µg/mL), *S. aureus* (128 µg/mL), *S. epidermidis* (8 µg/mL), *S. faecalis* (64 µg/mL).	[62]
	*P. aeruginosa* 47T2 MIC: dilution method	Antibacterial activity against Gram-negative bacteria: *A. faecalis* (64 µg/mL), *B. bronchiseptica* (128 µg/mL), *E. aerogenes* (4 µg/mL), *E. coli* (64 µg/mL), *K. pneumoniae* (0.5 µg/mL), *P. aeruginosa* (256 µg/mL), *S. thyphinurium* (128 µg/mL), *S. marcescens* (8 µg/mL). Antibacterial activity against Gram-positive bacteria: *A. oxidans* (128 µg/mL), *B. subtilis* (16 µg/mL), *M. luteus* (64 µg/mL), *M. phlei* (128 µg/mL), *S. aureus* (32 µg/mL), *S. epidermidis* (32 µg/mL), *C. perfringens* (128 µg/mL).	[63]
	*P. aeruginosa* BM02 MIC: microdilution method	Antibacterial activity against *S. aureus* and *E. faecium* (50 µg/mL)	[77]
	*P. aeruginosa* PAO1 MIC: Microdilution method	Antibacterial activity against *Cutibacterium acnes* (MIC: 15.62 µg/mL; MBC: 31.25 µg/mL)	[78]
	*P. aeruginosa* MR01 Diffusion test	Inhibition diameters (0.3 mg of biosurfactant): *B. cereus* (30 mm), *E. coli* (0 mm), *S. aureus* (14 mm), *S. epidermidis* (15 mm), *P. aeruginosa* (0 mm).	[79]
	*P. aeruginosa* DR1 Diffusion test	Mycelial growth inhibition: 60.46% (9 µg) *M. phaseolina*, 55% (12 µg) *F. oxysporium*, and 63.63% (13.5 µg) *P. nicotianae*.	[80]
	*P. aeruginosa* B5 MIC: microdilution method	Antifungal activity against *P. capsici* (10 µg/mL); *C. cucumerinum* and *C. orbiculare* (25 µg/mL); *C. destructans*, *C. kikuchii*, and *M. grisea* (50 µg/mL).	[81]
SLs	*Candida* sp. = AH62 MIC: microdilution method	Antimicrobial activity against: *B. subtilis* (2 mg/mL), *S. aureus* (1 mg/mL), *E. coli* and *P. aeruginosa* (4 mg/mL).	[82]
	*S. bombicola* MIC: microdilution method	Antimicrobial activity against *S. aureus* (31.25 µg/mL) and *L. monocytogenes* (62.50 µg/mL).	[83]
	*C. tropicalis* RA1 MIC: microdilution method	Antibacterial activity against *E. coli* (1000 µg/mL), *L. monocytogenes* (500 µg/mL), *S. aureus* (250 µg/mL).	[84]
	*R. babjevae* YS3 MIC: microdilution method	Antifungal activity against *T. mentgrophytes* (1 mg/mL—62% of inhibition); (4 mg/mL—100% of inhibition)	[85]
	*C. bombicola* ATCC 22214 MBEC: microdilution method	% Cell survival for *S. aureus*: 9.62% (6 µg/mL), 1.03% (8 µg/mL), 0.34% (10 µg/mL); for *B. subtilis*: 91.04% (0.6 µg/mL), 57.41% (0.8 µg/mL), 5.25% (1.0 µg/mL); for *E. coli*: 58.01% (10 µg/mL), 34.09% (20 µg/mL), 2.05% (30 µg/mL); for *P. aeruginosa*: 8.77% (1 µg/mL), 2.19% (3 µg/mL), 0.31% (5 µg/mL); for *C. albicans*: 10.34% (25 µg/mL), 10.34% (50 µg/mL), 6.89% (75 µg/mL).	[86]
Glycolipids	*S. saprophyticus* SBPS *15* Diffusion test	Antimicrobial activity against *K. pneumoniae* (23 mm, 0.2 µg), *E. coli* (20 mm, 0.6 µg), *P. aeruginosa* (20 mm, 1.6 µg), *V. cholerae* (18 mm, 3.2 µg), *B. subtilis* (15 mm, 2.4 µg), *S. paratyphi* (13 mm, 1.6 µg), *S. aureus* (11 mm, 0.6 µg). Antifungal activity against *C. neoformans* (22 mm, 1.6 µg), *C. albicans* (21 mm, 1.6 µg), *A. niger* (15 mm, 0.8 µg).	[87]
Surfactins	*B. circulans* Diffusion test	Zones of inhibition (1000 µg/mL of biosurfactant): *M. flavus* (17.00 mm), *B. pumilis* (15.33 mm), *M. smegmatis* (16.00 mm), *E. coli* (14.66 mm), *S. marcescens* (14.00 mm), *P. vulgaris* (10.66 mm), and *A. faecalis* and *K. aerogenes* (12.00 mm).	[76]
	*B. circulans* MIC: microdilution method	Antimicrobial activity against *M. flavus* (200 µg/mL), *B. pumilis* (30 µg/mL), *M. smegmatis* (50 µg/mL), *E. coli* (40 µg/mL), *S. marcescens* (30 µg/mL), *P. vulgaris* (10 µg/mL), *A. faecalis* (10 µg/mL), *K. aerogenes* (80 µg/mL).	[76]
	*B. velezensis* H3 Diffusion test	Zones of inhibition (100 µg/mL of biosurfactant): *C. albicans* (14 mm), *P. aeruginosa* (14 mm), *S.aureus* (11 mm), *K. peneumoniae* (10 mm).	[88]
	*B. subtilis* Diffusion test	Percentage of growth inhibition of *A. flavus* (%) with different concentrations of surfactins: 20 mg/L—36%, 40 mg/L—54%, 80 mg/L—84%, 160 mg/L—100%.	[89]
Surfactins and Fengycin	*B. subtilis fmbj* MIC: microdilution method	Antimicrobial activity against *B. cereus*: 156.25 μg/mL.	[90]
Fengycin	*B. thuringiensis* MIC: microdilution method	Antimicrobial activity against *C. albicans* and A. *niger* (15.62 µg/mL); *S. epidermidis* and *E. coli* (1000 µg/mL).	[91]
Iturins	*B. subtilis* K1 MIC: microdilution method	Iturin was more potent against *A. niger* and *A. brunsii* (2.5 μg/mL).	[92]
Lipopeptide	*B. cereus* Diffusion test and MIC: microdilution method	Zones of inhibition with 30 mg/mL of biosurfactant against *S. aureus* (20.2 mm), *E. coli* (20.2 mm), *P. aeruginosa* (16.0 mm), *K. pneumoniae* (15.0 mm), *C. albicans* (12.8 mm), *A. flavus* (11.4 mm). Antimicrobial activity against *S. aureus* (0.5 mg/mL), *E. coli* (1.04 mg/mL), *P. aeruginosa* (2.08 mg/mL), *K. pneumoniae* (4.16 mg/mL), *C. albicans* (7.6 mg/mL), *A. flavus* (7.6 mg/mL).	[93]

The proposed mechanism of action for rhamnolipids involves the insertion of their acyl tails into the lipid membrane, resulting in subsequent membrane disruption and increased permeability [34,94]. For instance, rhamnolipids, produced by *P. aeruginosa* AT10 with soybean oil refinery waste, have demonstrated activity against Gram-positive and Gram-negative bacteria [61,62]. Various bacterial strains were examined, with particular focus on those most relevant to human health. Rhamnolipids exhibited a MIC of 128 µg/mL against *S. aureus*, while against *S. epidermidis*, it showed a MIC of 8 µg/mL. When tested against *Enterococcus faecalis* and *Escherichia coli*, these mBSs displayed a MIC value of 32 µg/mL [62].

Moreover, rhamnolipids from *P. aeruginosa MR01* produced from a medium where glucose was used as a carbon source demonstrated antimicrobial activity through the disk susceptibility test after 16–18 h at 35 °C [79]. Although three different concentrations of rhamnolipids were tested (10, 20, and 30 mg/mL), the results showed that bacterial growth inhibition was not concentration-dependent. On one hand, the produced rhamnolipids were active against *Bacillus cereus* (30 mm), *S. aureus*, and *S. epidermidis* (15 mm), but on the other hand, they did not present antimicrobial activity against *E. coli* and *P. aeruginosa* (0 mm zone of inhibition) under the tested conditions [79].

Different concentrations of NaCl and pH values may affect the antimicrobial activity of rhamnolipids [95,96,97]. Ferroni Passos et al. (2024) evaluated the combined effect of pH and NaCl on the activity of commercial rhamnolipids against both planktonic and biofilms of *Listeria monocytogenes*. The antimicrobial activity is dependent on the pH and NaCl concentration of the medium and can be related to the type and size of the molecular aggregates. The tested biosurfactants were effective at pH 5 on planktonic and sessile cells, and the bactericidal efficiency was enhanced by the addition of 5% NaCl. When the pH was higher than 6, the effect of the salt was more evident and the antibacterial activity increased significantly. The planktonic cells were eliminated at pH 7.0 only when NaCl was present while MBIC varied from >2500.0 mg/L (RL) to 39.0 mg/L (RL + 5% NaCl), resulting in a 5 log decrease in biofilm viability [97].

Additionally, rhamnolipids have antifungal activity against some fungi [98]. This activity was demonstrated when using the diffusion method through measuring the inhibition in the diameter of mycelial growth containing the biosurfactant, compared to the control plate (without the biosurfactant). Although rhamnolipids did not present activity against *Sclerotium rolfsii*, they were able to inhibit mycelial growth (~60%) against *Macrophomina phaseolina*, *Fusarium oxysporium*, and *Phytophthora nicotianae* [80]. Moreover, Yan et al. demonstrated an inhibitory effect of 40.19% on the growth of *Alternaria alternata* when treating cherry tomatoes with a rhamnolipid solution (250 μg/mL) [99].

Other glycolipid biosurfactants with antimicrobial activity are sophorolipids, which seem to present the same mechanism of action as rhamnolipids. Sophorolipids act by modifying the hydrophobic properties of bacterial surfaces, leading to the disruption of membrane integrity and cell death [40,41]. When studying the antibacterial effect of sophorolipids produced by *S. bombicola* ATCC^®^ 22214™, Silveira et al. observed that a concentration of 31.25 and 62.50 µg/mL was able to inhibit *S. aureus* and *Listeria monocytogenes*, respectively [83]. Additionally, sophorolipids produced by *Candida tropicalis RA1* lead to an MIC of 1000, 500, and 250 µg/mL against *E. coli*, *L. monocytogenes*, and *S. aureus*, respectively [84]. Dengle-Pulate and coworkers evaluated the antimicrobial activity of sophorolipids produced by *C. bombicola* ATCC 22214 and calculated the percentage of cell survival compared to the control. Authors observed that the bacterial survival reached was 0.34% (10 µg/mL) with *S. aureus*, 5.25% (1.0 µg/mL) with *B. subtilis*, 2.05% (30 µg/mL) with *E. coli*, 8.77% (1 µg/mL) and 0.31% (5 µg/mL) with *P. aeruginosa*, and 6.89% (75 µg/mL) with *C. albicans* [86].

The synergistic antimicrobial effect of sophorolipid esters and piscidins, host defense peptides from fish, was evaluated [100]. The combined mixture of shophorolipid-hexyl ester with a sub-inhibitory concentration of piscidins 1 and 3 promoted more than a two-fold increase in antimicrobial activity against *Bacillus cereus*. Some mechanistic features were suggested, including the binding of piscidines 1 and 3 to the sophorolipid aggregate, the synergistic accumulation of piscidin–sophorolipid assemblies on the membranes, and the higher disruption of the lipid bilayer when in the presence of piscidin–sophorolipid complexes [100].

Expanding the structural variety of sophorolipids and other microbial biosurfactants is an important step in promoting their future applications and biological activities. Aiming at diversifying the class of sophorosides produced by an engineered *S. bombicola*, Pala et al. synthesized twenty-four new derivatives, including sophoroside amines with different alkyl chains lengths (ethyl to octadecyl) and their quaternary ammonium salts [101]. Moreover, other chemically modified glycolipid biosurfactants were hydrogenated to achieve the fully saturated lipid counterparts. The antimicrobial activities of the microbially produced glycolipids and their corresponding new derivatives were evaluated against Gram-positive (*B. subtilis*, *S. aureus*, *Listeria monocytogenes*) and Gram-negative bacteria (*E. coli*, *P. aeruginosa*) and one yeast strain (*C. albicans*). It could be concluded that microbially produced sophorosides and their hydrogenated derivatives exhibited selective antimicrobial activity against the tested micro-organisms. However, a broad antimicrobial activity was observed for lactonic sophorolipids, sophoroside amines, and quaternary ammonium salts, with some derivatives displaying MI values as low as 0.0137 mg/mL [101].

Mannosylerythritol lipids (MELs) have also shown antimicrobial properties [102,103]. MELs produced by *P. aphidis* showed an MIC of 1.25 mg/mL against *B. cereus*. Furthermore, the study revealed a correlation between the MEL concentrations and the antibacterial effect [104].

Lipopeptides can also damage and penetrate the cell membrane. This was detected by Carrillo et al. when studying the interactions between surfactins and the phospholipid bilayer. The authors observed that surfactins strongly bond to the membranes of phospholipids, damaging their structure. The rise in surfactin concentration increased the flow of cellular material leading to faster permeability changes in the membrane and a consequently high rate of loss of internal content to the external medium [105].

Aiming to analyze the antimicrobial potential of surfactins, Das et al. studied the surfactins produced by *Bacillus circulans* and concluded that surfactins led to the bacteria growth inhibition of *Alcaligenes faecalis*, *Proteus vulgaris*, and *E. coli* [76]. The study was conducted through the disk diffusion test with a 1000 µg/mL biosurfactant solution. Inhibition zones of 14.66, 10.66, and 12.00 mm were observed for *E. coli*, *P. vulgaris*, and *A. faecalis*, respectively. Moreover, the MIC was determined to be 40 µg/mL for *E. coli* and 10 µg/mL for *P. vulgaris* and *A. faecalis* [46,76,94]. In addition, surfactins produced by *Bacillus velezensis strain H3* were active against *S. aureus*, *Mycobacterium*, *Klensiela peneumoniae*, *P. aeruginosa*, and *C. albicans*. When surfactins were used at a concentration of 100 µg/mL, the diameters of inhibition halos observed were 14 mm for *C. albicans* and *P. aeruginosa*, 11 mm for *S. aureus*, and 10 mm for *K. peneumoniae* [88].

To present higher antifungal activity, a higher concentration of surfactins must be used. This was observed by Mohammadipur et al. through the disc diffusion test when using different concentrations of surfactins produced by *B. subtilis.* The authors observed that a concentration of 20 µg/mL pointed to an *A. flavus* growth inhibition of 36% passing to 54% with a concentration of 40 µg/mL and to 84% with a concentration of 80 µg/mL. To reach complete inhibition, a concentration of 160 µg/mL was necessary [89].

Additionally, mBSs have been studied for their antiviral activity against enveloped viruses. Viruses have two main components: the viral genome (RNA or DNA) and the capsid. In the case of enveloped viruses, there is a third element, the outer membrane that surrounds the capsid. An enveloped virus uses the host cell’s membrane to build its own membrane [106,107]. Biosurfactants can inactivate these viruses through physicochemical reactions that cause alterations in the viral capsid, outer coating, and lipid envelope. Consequently, the viral membrane will be unable to remain intact, and important cellular content will be lost [108,109].

Thus, rhamnolipids produced by *Pseudomonas* sp. strain S-17 showed activity against herpes simplex virus type 1 and type 2, with MIC values of 14.5 μg/mL and 13 μg/mL, respectively [110,111]. Also, sophorolipids derived from *C. bombicola* when at a concentration of 3 mg/mL have demonstrated anti-HIV activity [108,109,112,113]. Regarding SARS-CoV-2 (COVID-19), which is an enveloped virus, studies reported that sophorolipids [111] and rhamnolipids [114] have demonstrated an ability to damage this virus. Their activity is related to the modification of structural elements causing difficulties in the interaction between viral proteins and host cells, leading to a break in the viral cycle [26].

Moreover, Yuan et al. observed that surfactins produced by *B. subtilis* can eliminate the proliferation of porcine epidemic diarrhea virus (PEDV) when at a concentration of 15 µg/mL and transmissible gastroenteritis virus (TGEV) at 50 µg/mL [115].

It is known that bacteria and fungi become more tolerant to foreign agents when under biofilm settings. Biofilms are networks of microbial populations fixed to a surface and covered by an extracellular matrix composed of proteins, lipids, and polysaccharides, forming a protective barrier [10]. It is extremely difficult for antimicrobial agents to penetrate biofilms and kill pathogens without disrupting the environment and allowing micro-organisms to spread further. For this reason, new strategies to stop biofilm formation are required. Some studies proved the ability of biosurfactant solutions to act in the formation of biofilms. These results, including the type of mBS-producing strains, testing method, and antibiofilm activity towards different strains are summarized in Table 4.

**Table 4:** Biosurfactants as antibiofilm agents.

mBS	Micro-Organism, Method, and Surface	Results	Ref.
RLs	*P. aeruginosa* DS10-129; crystal violet staining; silicone.	Microbial inhibition (%): *R. dentocariosa*, 60.9%; *S. epidermidis*, 53.1%; *S. salivarius*, 58,2%; *S. aureus*, 33.8%; *C. albicans*, 38.2%; *C. tropicalis*, 35.3%.	[116]
	*P. aeruginosa* 89; crystal violet staining and MTT assay; medical-grade silicone. *P. aeruginosa* JS29; Crystal violet staining; 96-well microtiter plates	Biofilm reduction with 0.12 to 2 mg/mL of biosurfactant: 68–89% for *S. aureus*; 44–96% for *S. epidermidis*. 90% inhibition of biofilm formation by *S. aureus* at 2 mg/mL of RL-Glu and 0.5 mg/mL of RL-Gly, while 0.5 mg/mL of both rhamnolipid disrupted 90% of the preformed	[117] [118]
SLs	*S. bombicola* MTCC 1910; colorimetrix XTT microscopy; 96-well microtiter plates.	*Candida albicans*, *Candida tropicalis*, and *Candida lusitaniae* biofilms were inhibited when SL concentration was 120 μg/mL.	[119]
Surfactins	*B. amyloliquefaciens* NS6; crystal violet staining; polystyrene surfaces.	The *S. mutans* adhesion was reduced by 94.8% with 80 mg/mL.	[120]
	*B. safensis F4*; crystal violet staining; glass.	Inhibition of the biofilm formation against *S. epidermidis*: 90% with 10 mg/mL and 80% with 5 mg/mL.	[121]
Glycopeptide	*L. agilis CCUG31450*; crystal violet staining; 96-well microtiter plates.	Antiadhesive activity (%) against *S. aureus*: 64.6% (10 mg/L); 50.3% (1 mg/mL)	[122]

Rodrigues et al. evaluated the anti-adhesive activity of rhamnolipids produced by *P. aeruginosa* DS10-129 on silicone rubber [116]. Using a rhamnolipid solution at 4 g/L, a 60.9%, 53.1%, 58.2%, 33.8%, 38.2%, and 35.3% biofilm inhibition was observed (through the crystal violet method) against *Rothia dentocariosa*, *S. epidermidis*, *S. salivarius*, *S. aureus*, *C. albicans*, and *C. tropicalis*, respectively [116].

Other assays have also proved the ability of biosurfactants to inhibit *Candida* species biofilms on 96-well microtiter plates. An example is presented in Haque and colleagues’ work when testing sophorolipids produced by *S. bombicola* MTCC 1910 [119]. The authors observed a reduction of 80% in biofilm viability for *C. albicans*, *Candida tropicalis*, and *Candida lusitaniae* when using a sophorolipid solution at 120 μg/mL [119].

Similarly, surfactins have reduced bacteria adhesion on glass slides. When produced by *B. safensis F4*, surfactins at a concentration of 10 mg/mL inhibited the formation of *S. epidermidis* biofilm by 90%. When the concentration was reduced by half, the inhibition decreased to 80% [121].

This inhibition of biofilm formation is an important issue in hospital settings. Although medical devices are life savers, their usage is associated with different types of infections, as there is a great propensity for microbial colonization on their surfaces. Therefore, it is essential to improve biomaterial surfaces to prevent microbial colonization [13]. Several strategies that have been developed to improve the antimicrobial properties of surfaces through the use of mBSs will be further addressed in Section 4.

## 4. Enhancing Antimicrobial/Antibiofilm Activity of Materials with Biosurfactants

Approaches to prevent biofilm formation can be passive, repelling bacteria through physical/chemical modifications, the so-called antifouling strategies, or active, through the coating of surfaces with antimicrobial agents, interfering with the biological pathways of micro-organisms. These antimicrobial coatings can be either release-based or contact-based. Release-based approaches that have been proposed include different active compounds such as antibiotics, antiseptics, nitric oxide, and silver. Among the most suggested contact-based approaches, quaternary ammonium compounds, chitosan, antimicrobial peptides, and enzymes have been used [13,14,123]. Although the compounds mentioned are the most commonly used in antimicrobial strategies, biosurfactants should not be excluded since they can be used through these different strategies.

### 4.1. Release-Based Antimicrobial Approaches

**Figure 4:**
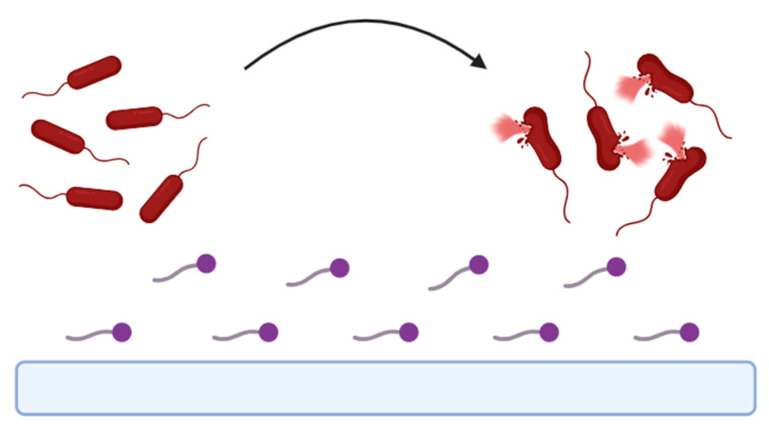
Representative scheme of release-based coatings. Antimicrobial compounds (presented in purple) will be released from biomaterial and promote micro-organism’s (presented in pink) membrane disruption and their death in surrounding environment and further prevent its attachment to biomaterial surface.

This strategy consists of the release of BSs from the surface of a biomaterial that will act on nearby bacteria, preventing their deposition (Figure 4). In this context, the most used approach is the adsorption of BSs to different biomaterial surfaces [13].

Aiming to create a new coating on titanium medical devices, Tambone et al. added a rhamnolipid (RL) solution (4 mg/mL) to previously polished, cleaned, and dried titanium discs [124]. This coating was based on the physical absorption of rhamnolipids to the titanium surface after 24 h of contact with the mBS solution. When comparing titanium surfaces coated with RLs with control titanium plates, the crystal violet staining method revealed a biofilm inhibition of 98.6% for *S. aureus* and 54.1% for *S. epidermidis* [124]. Additionally, lipopetides isolated from *B. subtilis* ATCC 19,659 were evaluated for their antimicrobial and antibiofilm efficacy on a titanium surface for dental implants. The studied biosurfactant exhibited antibiofilm activity for *S. aureus* and *S. sanguinis* with a 54% growth inhibition (MIC of 15.7 µg/mL) [125].

Moreover, under the scope of metallic surfaces coated with biosurfactants, Nitschke et al. demonstrated that the immersion of stainless steel surfaces in an aqueous solution of surfactins reduced the number of adhered species of *L. monocytogenes* (with a reduction from 7.9 to 5.7 log CUF/cm^2^) and *Enterobacter sakazakii* (with a reduction from 5.3 to 4.5 log CUF/cm^2^). Moreover, the authors similarly performed the same experiment with polypropylene and observed a decrease in adhered *L. monocytogenes* (with a reduction from 6.2 to 5.6 log CUF/cm^2^), *E. sakasakii* (with a reduction from 6.2 to 5.4 log CUF/cm^2^), and *Salmonella enteritidis* (with a reduction from 6.1 to 5.8 log CUF/cm^2^) [126].

Regarding silicone, a biomaterial commonly used in the production of medical devices, Pontes et al. studied the effect of adsorbing a mixture of sophorolipids (acidic and lactonic) produced by *S. bombicola* onto silicone strips. The silicone surfaces were first immersed in sophorolipid solutions at different concentrations to promote their adsorption. After 24 h of bacterial contact with adsorbed samples, the formed biofilm on the silicone surfaces was assessed through the crystal violet staining method. The results showed a 2 and 3 log CFU/cm^2^ reduction of *S. aureus* biofilm when sophorolipid solutions of 0.38 mg/mL and 1.5 mg/mL were used [127]. Later, the same group performed the same experiment but with a mixture of lactonic sophorolipids produced by *S. bombicola* and observed a 4 log CFU/cm^2^ reduction of the *S. aureus* biofilm on the silicone surface when the lactonic sophorolipid mixture used was at 0.38 mg/mL [128]. Lactonic sophorolipids are known for their higher antimicrobial activity; thus, the purification of the crude mixture to achieve only a lactonic mixture led to an increase in antibiofilm activity.

Besides the adsorption of biosurfactants onto surfaces, their incorporation into nanoparticle delivery systems has also been proposed for biofilm formation prevention through release approaches. Bettencourt et al. used chitosan nanoparticles incorporated with rhamnolipids from *P. aeruginosa* and observed an antimicrobial effect against *S. aureus* with an MIC of 130 μg/mL (microdilution method). Moreover, nanoparticles incorporated with rhamnolipids were capable of a 99% inhibition of *S. aureus* biofilm formation on medical-grade silicone segments [129]. Additionally, the achieved particles were positively charged, which may come as an advantage considering that *S. aureus* membranes are negatively charged, leading to greater electrostatic interactions between both and more efficient cell disruption [129]. Other authors have also developed rhamnolipid–chitosan nanoparticles and observed that their antimicrobial/antibiofilm activity against *S. aureus* (14 μg/mL), *S. epidermidis* (7 μg/mL), and *Klebsiella oxytoca* (116 μg/mL) was higher when compared to rhamnolipid-free chitosan NPs. Concerning biofilms, it is believed that the strong interaction of chitosan with bacteria allows nanoparticles to accumulate on their surface, allowing antimicrobial agents to diffuse into the bacterial colonies [130].

Another possible biosurfactant release strategy is the release from hydrogel coatings. An example is medical-grade silicone functionalization with a sophorolipid–hydrogel coating to enhance its antimicrobial activity. This was observed by Narciso et al. [131] when studying the suitability of a sophorolipid–chitosan hydrogel 3D-printed coating for improving the antibiofilm activity of medical-grade silicone. The achieved coatings presented cytocompatibility under the tested conditions and the sophorolipid–chitosan coatings reached an almost 2 log CFU/cm^2^ inhibition of *S. aureus* biofilm formation.

Da Silva et al. evaluated the antibiofilm activity of a cationic rhamnolipid derivative containing arginine, both alone and incorporated into a gel prepared with Pluronic F-127, against biofilms of fluconazole-resistant *C. albicans* (FRSA) and methicillin-resistant *S. aureus* (MRSA) in impregnated peripheral venous catheters [132]. The rhamnolipid derivative exhibited antimicrobial activity against planktonic cells of *Candida* spp. (with MIC values of 7 to 21 µg mL^−1^) and S. aureus (with MIC values of 5 to 11 µg mL^−1^) strains, being more effective than fluconazole and oxacilin. Moreover, it also significantly reduced cell viability in resistant micro-organisms’ biofilms (FRSA and MRSA), with a reduction of up to 81.8%. Additionally, the surfactant gel or pure solution was impregnated into peripheral venous catheters, and the ability to inhibit the development of biofilms was further investigated. It was shown that after 28 days, the cationic biosurfactant almost completely inhibited the growth of the FRCA/MRSA mixed biofilms and the antibiofilm activity on these medical devices remained unchanged [132].

Despite the positive results described, to reach a long-term activity, longer-lasting approaches such as the bonding of antimicrobial agents to biomaterials surfaces must be applied. Some of those strategies will be further discussed.

### 4.2. Contact-Killing Antimicrobial Approaches

When antimicrobial compounds are covalently bonded to surfaces, effective contact-killing approaches can be achieved. This can also be accomplished using mBSs, which interact with and disrupt the micro-organisms’ membranes, leading to their death [13]. Covalent bonds between biosurfactants and biomaterial surfaces can form through some biosurfactant functional groups such as carboxyl or amine groups and some examples will be further presented [133].

**Figure 5:**
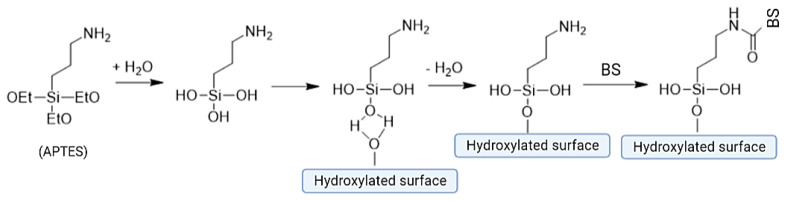
Reaction scheme of silanization: hydrolysis, hydrogen bonding, condensation.

The functionalization of rhamnolipids produced by *P. aeruginosa* on a polydimethylsiloxane (PDMS) surface achieved by Dardouri et al. is an example of a contact-killing approach. The authors first performed PDMS surface oxidation using a “piranha solution” (sulfuric acid and hydrogen peroxide) [134,135]. Next, silanization takes place, which is a process where it is possible to cover a wide range of hydroxylated surfaces, such as glass and metal, with alkoxysilane molecules. Thus, after surface oxidation, the material was submerged in a silane solution, i.e., (3-aminopropyl) triethoxysilane (APTES), to reach a surface rich in amine groups, capable of creating a bond with the biosurfactant’s carboxyl group (Figure 5). Following hydrolysis, the silane connects to the hydroxylated surface by hydrogen bonding, and finally, condensation occurs, covalently bonding the silane to the surface [133,136].

**Figure 6:**
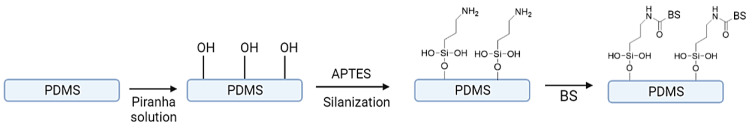
Reaction scheme—oxidation, silanization, peptide bond.

Finally, rhamnolipids were then converted into N-hydroxysuccinimide esters by sequential reaction with N-(3-Dimethylaminopropyl)-N′-ethylcarbodiimide hydrochloride (EDC) and N-hydroxysuccinimide (NHS) to promote its bonding to the APTES amine group present at the PDMS surface [134,135]. This functionalization strategy makes the surface less hydrophobic (Figure 6).

When evaluating the antimicrobial activity of PDMS functionalized with rhamnolipids, Dardouri et al. [134] observed a log (CFU/cm^2^) reduction of 4.20 (99.99%) against *S. aureus*, 1.17 log (93.26%) against *S. epidermidis*, and 0.95 log (88.78%) against *C. albicans*. Biofilm reduction was also evaluated in co-culture assays (towards the same micro-organisms), with results between a 1 and 2 log reduction [134].

In another study from the same authors [137], identical methods for rhamnolipids functionalization onto PDMS were used but surface activation was performed through a different method. Plasma activation was used to reach oxidized surfaces instead of a “piranha solution” [135,137] since plasma treatment leads to oxygen radical formation. A reduction of *S. aureus* biofilm of 2.4 log (CFU/cm^2^) and 1 log (CFU/cm^2^) against *S. epidermidis* was observed [137].

**Figure 7:**
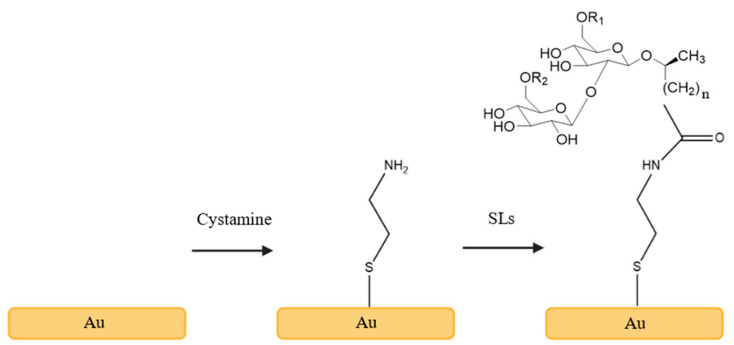
Functionalization of gold surfaces with acidic sophorolipids (A-SLs).

Regarding sophorolipid functionalization, Valotteau et al. reported a method for functionalizing Au surfaces with sophorolipids [138,139]. First, gold substrates were immersed in an ethanolic solution of cystamine. Next, the carboxylic acid end of the SLs was activated using a mixture of EDC and NHS to facilitate the reaction with the amine group of cystamine previously bound to the surface (Figure 7). This functionalization was able to damage the membrane of Gram-positive bacteria (*E. faecalis*, *S. epidermidis*, *Streptococcus pyogenes*) and Gram-negative bacteria (*E. coli*, *P. aeruginosa*, *Salmonella typhimurium*) [138,139].

### 4.3. Antifouling Approaches

Besides being essential for contact death approaches, it is most likely that covalent bonding of BSs to surfaces will add anti-adhesive properties.

Dardouri et al. studied the functionalization of rhamnolipids on a PDMS surface through the two previously mentioned methods [134,135,137] and observed that the wettability of the surface increased since the water contact angle decreased. Thus, functionalization of PDMS with rhamnolipids modifies the physicochemical characteristics of PDMS, making it less hydrophobic, disfavoring the adhesion of some bacteria and/or fungi [134].

Moreover, Mendes et al. also evaluated the anti-adhesive activity of silicone surfaces functionalized with sophorolipids produced by *S. bombicola*. The acidic sophorolipids were converted to esters by N-(3-dimethylaminopropyl)-N’-ethylcarbodiimide hydrochloride. Then, the solution was introduced into the freshly aminated silicone substrate, forming a bond between the amine and the carbonyl group. This surface amination was achieved through surface oxidation and silanization, a strategy identical to that outlined in Figure 5. In this case, only the acidic sophorolipids can bind to the surface, because the lactonic ones do not present the free carboxylic group capable of covalently bonding to the free amine on the surface. The results showed that after covalent functionalization, the colonization values of micro-organisms on the surface decreased to 20% [128].

Valotteau et al. also obtained antiadhesive surfaces when carrying out the functionalization of deacetylated acidic *cis* C18 sophorolipids (SL) provided by Bio Base Europe Pilot Plant and deacetylated acidic fully saturated C18 SL through grafting onto flat polycrystalline gold substrates via a self-assembled monolayer of short aminothiols (Figure 5). The authors concluded that the addition of the glycolipid (1 to 100 mg/mL) not only interacts with the bacterial membranes but also reduces the likelihood of *S. aureus* and *E. coli* adhesion to the surfaces [140].

**Figure 8:**
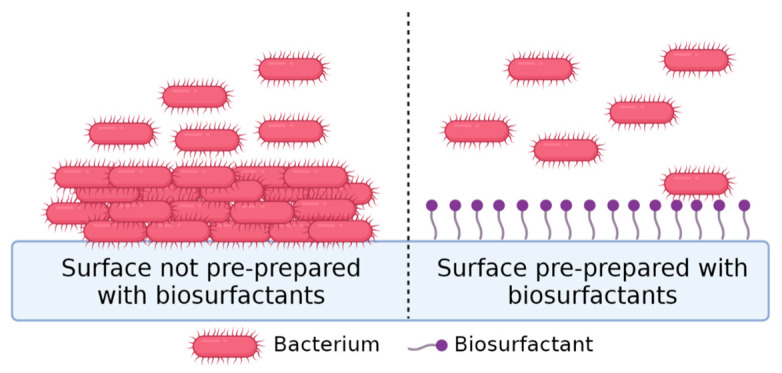
Representative scheme of bacteria adhesion when adding biosurfactants to surfaces.

Antifouling approaches repel bacteria through physical/chemical modifications, preventing biofilm formation. In this case, the micro-organisms are not killed, only prevented from depositing/attaching on surfaces. Figure 8 illustrates what happens when a surfactant is bound to a hydrophobic surface. The hydrophobic tail is disposed on the surface and the polar head is oriented towards the aqueous medium; in this way, the surface will increase its hydrophilic character, decreasing the interfacial tension between the surface and the water and the deposition of bacteria [14,74,141,142].

## 5. Structure–Activity Relationship (SAR) of Biosurfactants

Understanding the chemical structures of mBSs and their corresponding mechanisms of action is crucial for developing interesting antimicrobial applications. Table 5 summarizes the structure–activity relationships of different types of mBSs.

Regarding rhamnolipids, researchers have studied how a structural difference between one or two rhamnose sugar units can affect the antibacterial activity. Zhao et al. observed that mono-rhamnolipids (mono-RLs) showed a greater inhibition diameter for all bacteria and fungi when compared to di-rhamnolipids (di-RLs), when using the disk diffusion test towards five different micro-organism strains. Another test was carried out in the same study to confirm the antimicrobial activity of RLs by calculating IC_50_ values of mono-RLs and di-RLs through the OD600 in liquid culture. The IC_50_ value of mono-RLs was less than 5 mg/L, while for the di-RLs it was 10 mg/L, which led to the conclusion that rhamnolipids with only one rhamnose unit in their structure showed higher activity [143]. On the other hand, other studies have demonstrated that di-RLs present greater antifungal activity [64,144].

The lactonic variants of sophorolipids exhibited more pronounced biological activities, including antibacterial, fungicidal, spermicidal, and anticancer effects [145]. In contrast, acidic sophorolipids have been reported to possess slightly higher antiviral activity [146]. This demonstrates that their chemical structure significantly influences their properties. Mendes et al., when assessing the influence of chain unsaturation in diacetylated lactonic sophorolipids produced by *S. bombicola* on antimicrobial activity against *S. aureus*, observed that C18:0 and C18:1 showed a lower MIC (50 μg/mL) compared to C18:2 and C18:3 (200 μg/mL), suggesting that one or two double bonds in the chain of lactonic sophorolipids enhanced their antimicrobial activity [128]. Furthermore, the degree of acetylation also affects sophorolipids’ antimicrobial properties. The MIC value against *B. cereus* decreased from 25 µM to 12 µM when comparing monoacetylated to diacetylated sophorolipids, suggesting that higher acetylation levels increase the antimicrobial activity [147].

**Table 5:** Analysis of the structure–activity relationships of different types of mBS.

mBS	Structure	Activity						Ref.
Rhamnolipids (*P. aeruginosa*)		Antimicrobial Activity against *B. Wiedmannii*						[143]
		Inhibition zone (mm)		Inhibition rate (%)		IC_50_ (mg/L)		
	Mono-	30.7 ± 2.5		98.9		<5		
	Di-	20.3 ± 1.5		97.8		10		
		IC50 (μg/mL) against *Oomycetes*, *Ascomycota*, and *Zygomycetes*						[144]
	Mono-	70.8–1271.0						
	Di-	7.0–114.5						
		Growth inhibition (%) for *A. carbonarius*						[64]
	Mono-	30.2						
	Di-	33.1						
Sophorolipids (*S. bombicola*)		MIC (μg/mL) for *S. aureus*						[128]
	C18:2 DLSL	200						
	C18:1 DLSL	50						
	C18:0 DLSL	50						
Sophorolipids (synthetics)		MIC (µM) for *B. Cereus*						[147]
	Monoacetylated	25						
	Diacetylated	12						
Mannosylerythritol Lipids (synthetics)		MIC (μg/mL) for *M. luteus*						
		MEL-A	MEL-B		MEL-C		MEL-D	[148]
	C6	128	>128		128		128	
	C8	32	16		32		32	
	C10	8	10		10		8	
	C12	128	128		128		64	
	C14	128	128		128		128	
		Anticancer activity						
Rhamnolipids (*P. aeruginosa*)		HL-60	BV-173		SKW-3		JMSU-1	[145]
	Mono-	67	50		54		60	
	Di-	77	82		108		140	
Sophorolipids (*S. bombicola*)		IC50 (μg/mL) of HeLa cancer cells						[146]
	C18:2 DLSL	476.46						
	C18:1 DLSL	12.23						
	C18:0 DLSL	30.24						
	C16:1 DLSL	62.78						
	C16:0 DLSL	62.95						
Surfactins (*B. subtilis*)		IC50 (μg/mL) of Bcap-37 cancer cells						[149]
	C13	60.81						
	C14	41.26						
	C15	29.7						

Sophorolipids’ (SLs’) anticancer activity has also been studied. When evaluating several diacetylated lactonic sophorolipids against human cervical cancer cells, it was found that the IC_50_ values for C16:0 and C16:1 (62.95 and 62.78 μg/mL, respectively) were higher than those for C18:0 and C18:1 (30.24 and 12.23 μg/mL, respectively). This indicates that longer, more lipophilic chains result in improved anticancer activity. Furthermore, the degree of unsaturation also plays a crucial role. Comparing the IC_50_ values of C18:0 (30.24 μg/mL), C18:1 (12.23 μg/mL), and C18:2 (476.46 μg/mL) revealed that a single double bond in the chain is ideal for achieving higher anticancer activity [146]. Moreover, Ribeiro et al. suggested that an increase in the degree of unsaturation in the SL molecule results in less cytotoxicity against MDA-MB-231 cells. Furthermore, the cytotoxic effect against MDA-MB-231 cells was greater with lactonic SLs compared to acidic SLs [65].

A structure–activity relationship analysis of MELs has also been conducted [148]. Twenty MEL molecules with different alkyl chain lengths (C6, C8, C10, C12, and C14) were evaluated. MELs with a ten-carbon chain were more effective against *M. luteus*, showing MIC values of 8 μg/mL (MEL-A and MEL-D) and 10 μg/mL (MEL-B and MEL-C). It was also possible to conclude that very short or very long chains (C6 and C14, respectively) presented similar and unsatisfactory results [148].

Finally, differences in the length of the surfactin’s carbon chain also affect its biological activity. The level of a surfactin’s penetration into the cell membrane is directly proportional to the chain length [149]. Liu et al. demonstrated that surfactins produced by *B. subtilis HSO121* with a C15 chain presented greater antitumor activity towards Bcap-37 cells, showing an IC_50_ of 29.7 μg/mL, while with a C13 or C14 chain, surfactins presented an IC_50_ of 60.81 μg/mL and 41.26 μg/mL, respectively [149]. Moreover, inactivation of enveloped viruses (VSV, SFV, and SHV-1) with surfactins was also more effective with the C14 and C15 isoforms than for C13 [150,151].

## 6. Conclusions and Future Perspectives

Biosurfactants have been the subject of intense research due to their biocompatibility, biodegradability, low toxicity, and antimicrobial and anticancer properties. Among biosurfactants with antimicrobial activity are rhamnolipids, sophorolipids, trehalose lipids and mannosylerythritol lipids; the best-known lipopeptide surfactins, iturins, and fengycin; and, finally, emulsan, liposan, and alasan. Due to their mechanism of action being associated with micro-organisms’ membrane interference and disruption, their activity towards Gram-positive bacteria is more evident. Nevertheless, activity towards Gram-negative bacteria and fungi has also been reported. 

The antimicrobial activity of biosurfactants can be explored in the context of infection prevention, namely infections associated with medical devices. In this context, one of the most promising approaches can be achieved by preparing these surfaces against the deposition of micro-organisms. A long-lasting methodology is the functionalization of surfaces through covalent bonds with biosurfactants. Besides leading to the death of pathogens through contact killing, this strategy will also repeal their attachment through physicochemical interactions. In addition, simpler strategies can also be used, without surface preparation, based on the absorption of biosurfactants onto the surface to be released, eliminating micro-organisms in the vicinity. Moreover, among release strategies are rhamnolipid nanoparticles and polymeric hydrogels loaded with sophorolipids that also work as antimicrobial and antibiofilm approaches.

Improved antimicrobial results may be achieved if the structure–activity relationship of these molecules is known. Few studies have been conducted in that area, and some do not present corroborative results. In some studies, di-rhamnolipids showed better antifungal activity, whereas mono-rhamnolipids revealed higher antibacterial activity. Diacetylated lactonic sophorolipids such as C18:0 and C18:1 present higher antimicrobial activity. Mannosylerythritol lipids presenting a ten-carbon chain show higher antimicrobial activity and a surfactin’s penetration into the phospholipid cell membrane is directly proportional to its chain length.

Research on biosurfactants is increasing due to their promising applications in several areas, including the pharmaceutical, cosmetics, agriculture, oil, detergent, and food industries and environmental bioremediation, among others. However, their commercial success remains a challenge despite the high number of academic studies. Therefore, future research is mandatory to maximize the potential of biosurfactants and promote broader industrial applications. Some directions for future research may include the following: (i) The development of sustainable and economical production strategies, using low-cost substrates, waste residues or immobilized cell systems to reduce production costs. (ii) The study of new microbial sources for novel biosurfactants with improved stability, bioactivity, and specificity. (iii) The use of genetic engineering and biotechnology to modify microbial strains to achieve higher production yields or enhanced properties. (iv) The promotion of further comprehensive studies on toxicity, environmental persistence, and long-term ecological effects. (v) The evaluation of the use of biosurfactants in environmental applications such as oil remediation, heavy metal removal, and soil detoxification; (vi) Addressing regulatory hurdles and large-scale production challenges through industry collaboration towards clear guidelines and production standards.

Regarding the emergent need for novel antimicrobial solutions, future studies could be focused on the association of biosurfactants with other antimicrobial molecules not prone to resistance to reach greater antimicrobial activity. Moreover, the association of different antimicrobial approaches such as contact killing and release-based approaches may lead to a boost in antimicrobial/antibiofilm activity. Additional efforts must also be applied to the production and purification of biosurfactants to profitably reach purified compounds that present higher antimicrobial activity. This will certainly enhance the antimicrobial/antibiofilm activity of biosurfactants and increase their applications such as in the field of medical-device-related infection prevention.

## Figures and Tables

**Table 1:** Summary of the mBSs, considering their classes, the producing micro-organism, and characteristic chemical structures.

Class of mBS	Type	Main Producer Micro-Organism	Structure
Glycolipids	Rhamnolipids	*Pseudomonas aeruginosa*	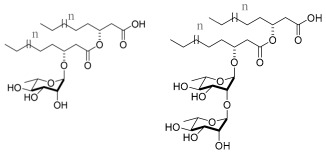
	Trehalose Lipids	*Rhodococcus erythropolis*	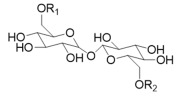 R_1_ = R_2_ = OCCH(CH_2_)_n_CH_3_CHOH(CH_2_)_m_CH_3_; R_1_ = OCCH(CH_2_)_n_CH_3_CHOH(CH_2_)_m_CH_3_ and R_2_ = H
	Sophorolipids	*Candida bombicola*	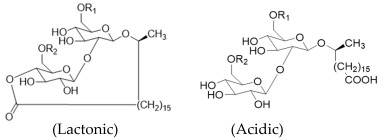 R_1_ = R_2_ = H; R_1_ = H and R_2_ = OAc; R_1_ = OAc and R_2_ = H; R_1_ = R_2_ = OAc
	Mannosylerythritol Lipids	*Pseudozyma antartica and Pseudozyma aphidis*	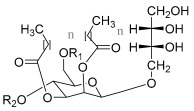 MEL-A: R_1_ = R_2_ = Ac; MEL-B: R_1_ = Ac R_2_ = H; MEL-C: R_1_ = H R_2_ = Ac; MEL-D: R_1_ = R_2_ = H
Lipopeptides and Lipoproteins	Surfactins and Iturins	*Bacillus subtilis*	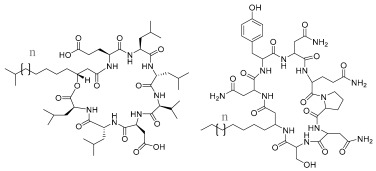
Phospholipids, Fatty acids, and Neutral Lipids	Phosphatidylethanolamine	*Acinetobacter radioresistens*	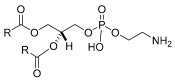
Polymeric Biosurfactants	Emulsan	*Acinetobacter calcoaceticus*